# Retardation Effects of Filter Mud in Molasses on Composite Silicate Cement

**DOI:** 10.3390/ma15113989

**Published:** 2022-06-03

**Authors:** Xun He, Hui Jiang, Xin Wan, Kequan Chen, Pingkai Ouyang

**Affiliations:** State Key Laboratory of Materials Oriented Chemical Engineering, College of Biotechnology and Pharmaceutical, Nanjing Tech University, Nanjing 210009, China; huijiang@njtech.edu.cn (H.J.); wanx0813@163.com (X.W.); kqchen@njtech.edu.cn (K.C.); ouyangpk@njtech.edu.cn (P.O.)

**Keywords:** biomass, biochemical engineering, chemical processes, molasses, retardation effect, biorefinery

## Abstract

The filter mud in molasses has a significant inhibitory effect on biological activity and cannot be utilised by organisms; therefore, before molasses are biotransformed, the filter mud will be separated and directly discarded in the environment. In this study, the filter mud was used as the retarder of cement concrete OPC 42.5 for the first time. It was found that when 0.2–0.8% filter mud was added to fresh cement concrete OPC PC 42.5, the hardening time of cement slurry was significantly prolonged due to the synergistic retarding effect of sugar, colloid and total cellulose in the filter mud. In addition, the compressive strength of cement concrete mixed with the filter mud in the early stage (<10 days), middle stage (10–100 days) and later stage (180 days) was significantly higher than that of cement concrete and cement concrete mixed with commercial asphalt lignosulfonate. These results showed that the filter mud in molasses could realise harmless and resource utilisation, which could promote the comprehensive utilisation of molasses.

## 1. Introduction

The main reason for cement setting and hardening was that mineral compositions, dicalcium silicate 2CaO·SiO_2_, tricalcium silicate (C_3_S) and tricalcium aluminate (C_3_A), of clinker conduct the hydrolysis or hydration reaction with water, and produce hydration products such as calcium hydroxide Ca(OH)_2_, hydrated calcium silicate (C-S-H), hydrated calcium aluminosilicate (C-A-S-H), etc. [1]. These hydration products, depending on various gravitational forces to overlap and bind, form a cement structure with a certain strength. The added retarder can postpone the rate of cement hydration products absorbing and condensing into a continuous reticular flocculation structure, postpone the cement hydration reaction and extend the concrete setting time, and make the fresh concrete maintain not only plasticity but also other properties over a longer period [2,3]. Currently, the commonly used retarders include small molecule sugar, inorganic boric acid (inorganic borate), organic lignosulfonate and its derivatives, hydroxycarboxylic acid (hydroxy carboxylate), etc. [4,5,6]. Inorganic electrolytes can discharge charged ions by ionisation in an aqueous solution and form a double electrode layer on the surface of the cement particle to prevent the cement particle from bonding with each other, which affects the precipitation nucleation process of Ca(OH)_2_ and C-S-H and the formation of C-A-S-H [1], thereby postponing cement setting and hardening. The mechanism of organic retarders of lignosulfonate and hydroxycarboxylic acid (hydroxy carboxylate) mainly lies in the following: (1) active groups such as the oxhydryl group (-OH), amino group (-NH_2_), and carboxyl group (-COOH) are contained in the molecular structure, in which the oxhydryl group can form an unstable clathrate with the free Ca^2+^ in the alkaline medium of cement hydration products, and retardation is caused by controlling the Ca^2+^ concentration in the liquid phase at the initial stage of hydration. With the progress of hydration, the unstable clathrate will automatically decompose, and hydration will continue; (2) -OH, -NH_2_, -COOH groups are easily associated with water molecules through hydrogen bonding, coupled with the hydrogen bond association between water molecules, and a solvated water film will be formed on the surface of cement particles [7]; thus, the direct contact between the cement particles is prevented, and the hydration process is hindered; (3) -OH groups can be absorbed on the surface of cement particles and form hydrogen bonds with O_2_− on the surface of hydration products [8], coupled with hydrogen bond association of the -OH groups with water molecules. Likewise, a solvated water film will be formed on the surface of cement particles [7]; thus, the hydration process of cement is inhibited, and with the increase in the number of -OH groups, retardation is gradually strengthened [9,10,11].

Sugar and sugar derivatives contain active -OH groups and can be converted into saccharic acid containing -COOH in an alkaline solution [12]. Sugar and saccharic acid can be absorbed either on the surface of cement particles and hydration products or in nucleation sites of Ca(OH)_2_ and C-S-H, thus hindering the setting and hardening of fresh concrete [13,14].

Molasses is a cheap waste of biomass, and it is commonly used for the biosynthesis of many chemical products; however, molasses was often solid–liquid separated first, then the sugar solution with high sugar concentration was utilised [15], and the filter mud obtained after solid–liquid separation was almost directly discarded [16]. Cane molasses showed good grinding aid to cement, which slightly prolonged the setting time of cement and could significantly increase the compressive strength at all ages [17]; however, the effect of filter mud in molasses on the performance of cement has not been reported. In this paper, filter mud in molasses left after centrifugation was directly used as a cement admixture alone and fully mixed with cement, aggregates, and mortar to prepare fresh concrete. Molasses raw materials, colloid, sugar solution, water-soluble pigment, and commercially available lignosulfonate were used as controls to compare the setting time, slump, bleeding rate and other properties of fresh concrete prepared by different mixing systems and to study the properties of hardened concrete, such as strength, water absorption, drying shrinkage, freezing and thawing, wetting and drying, and carbonation.

In this paper, filter mud, a significant biological inhibitory component in molasses, was successfully utilised as a resource for the first time, that is, it was used as a cement retarder, which was conducive to increasing the types of molasses-based products and improving the comprehensive utilisation rate of molasses.

## 2. Materials and Methods

### 2.1. Cement

Ordinary Portland cement OPC PC 42.5 was purchased from Shandong Linyi San’en Import and Export Co., Ltd. (Linyi, China). The physical properties and chemical composition of the cement are given in Table 1.

### 2.2. Aggregates

Two kinds of crushed particles, mainly consisting of dolomite were used as aggregates: aggregate 1 (maximum particle size 16 mm) and aggregate 2 (maximum particle size 38 mm). Natural sand (maximum particle size 2 mm) and crushed stone sand (maximum particle size 4 mm) were used as fine aggregates. The volume proportions of various components in the aggregate mixture were 26% of aggregate 1, 28% of aggregate 2, 36% of natural sand, and 10% of crushed stone sand.

### 2.3. Admixtures

**Figure 1:**
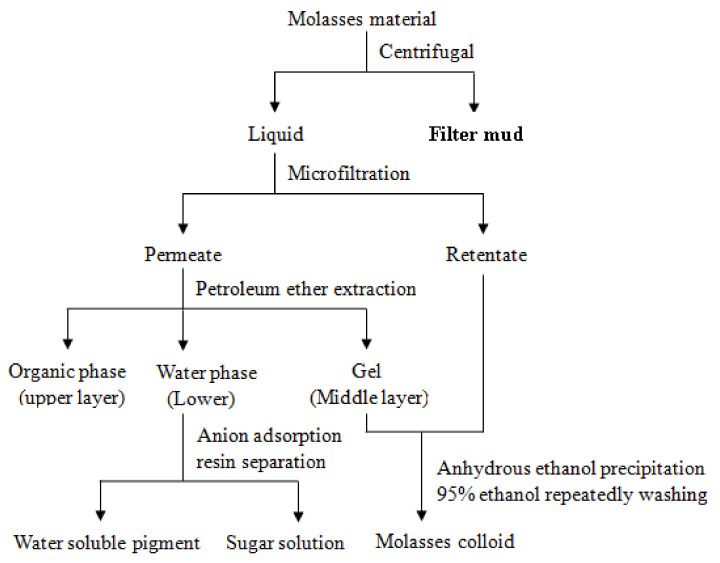
Separation steps of molasses raw material.

Admixtures include Ca-lignosulfonate, molasses raw materials, filter mud from molasses, molasses colloid, sugar solution and water-soluble pigment. The molasses raw materials were purchased from Shandong Jinan Sugar Company (Jinan, China); the filter mud from molasses, colloid, sugar solution and water-soluble pigment was obtained by a gradual separation of the molasses raw materials (as shown in Figure 1), and the content of each component is given in Table 2. All admixtures were formulated as a 40% (*w*/*w)* solution. Formaldehyde was added to the mixture to prevent fermentation.

### 2.4. Mortar and Concrete

The weight ratio of cement/sand/water in the mortar was 1:2.5:0.5. The cement content and *W/C* (*W/C*: mass ratio of water to cement per cubic meter of concrete mixture) for all concrete mixtures were the same: 320 kg·m^−3^ and 0.65, respectively. The weight ratio of cement/aggregate 2/aggregate 1/crushed sand/natural sand was 1:1.7:1.6:0.6:2.0. The mixture was mixed in a 6 L mixer for the concrete test (Type MY3000-6E, Wuhan Meiyu Instrument Co., Ltd., Wuhan, China) for 3 min.

### 2.5. Analysis

#### 2.5.1. Initial Setting Time and Final Setting Time

The initial setting time and final setting time of cement concrete were measured by a Vicat apparatus (Type DWZ-120, Hebei Dahong Laboratory Apparatus Co., Ltd., Shijiazhuang, China) according to the method specified in GB/T1346-2011. The sample of cement concrete was cured in a moisture curing case (Type HTD2128 Hebei Runchuang Technology Development Co., Ltd. Shijiazhuang, China), and the measurement was carried out 30 min after the sample was added to water. 

#### 2.5.2. Slump

According to the methods specified in GB/T 50081-2002 and GB/T 50080-2002, the slump of the fresh concrete sample was measured by a concrete slump tester (Type TLD-1, Hebei Dahong Laboratory Apparatus Co., Ltd.).

#### 2.5.3. Bleeding Rate

The seepage characteristics of fresh concrete were analysed according to the bleeding rate. The test was carried out in the following method: A 5 L cylinder (with an inner diameter of 185 mm and a height of 200 mm) with a cover was dampened by a wet cloth; the fresh concrete was loaded into the cylinder one time and vibrated on a vibrating table; the surface of the cylinder was plastered with a trowel, and the cover was affixed to prevent evaporation. The surface of the sample was approximately 20 mm lower than the edge of the cylinder. From the time after the plastering operation, the bled water was sucked with a suction tube one time every 10 min in the first 60 min and one time every 20 min thereafter until three successive sucking operations had no water available. In the first 5 min before every sucking operation, one side of the bottom was underlaid up approximately 20 mm to make the cylinder tilt for water suction. After the sucking operations, the cylinder was gently placed flat and covered. The water obtained was fed into a graduated cylinder with a plug, the total bled water was measured, and the bleeding rate B was calculated according to Formula (1).

(1) $$B=\frac{V_{W}}{(W/G)G_{W}}\times 100$$

where *V_W_* is the total bled water/g, *W* is the water consumption of fresh concrete/g, *G* is the total fresh concrete/g, and *GW* is the mass of sample/g.

#### 2.5.4. Compressive Strength

Compressive strength tests were carried out on cubic specimens of 15 × 15 × 15 cm^3^ at the ages of 1 d, 3 d, 7 d, 28 d, 90 d, 180 d and 200 d. The sample surface was compressed by a loading rate of 0.3–0.8 MPa·s^−1^ until the sample was near damage and started to deform, and the failure load was recorded.

#### 2.5.5. Others

Capillary tests were conducted for cubic specimens of 10 × 10 × 10 cm^3^ cut from concrete specimens mentioned above on the 180th day to determine the freezing–thawing and wetting-drying properties. The sample was preserved for 28 days in saturated lime water and then in a laboratory with a relative humidity of 65 ± 5% and a temperature of 22 ± 2 °C. The sample was frozen in air, thawed and wetted in water. A wetting-drying cycle of 24 h was conducted. The sample was dried for 4 h in air at a temperature of 22 °C and then dried for 20 h in a baking oven at a temperature of 50 ± 2 °C. The degree of qualitative change in cement concrete should be determined by resonance frequency tests, ultrasonic velocity tests, and weight loss tests. A carbonation test was conducted for the fresh concrete sample after hardening for 28 days in the conditions with a relative humidity of 65 ± 5% and a temperature of 22 ± 2 °C, with phenolphthalein used as a lime indicator.

Each of the above tests was conducted three times in parallel, and related data were recorded as the average ± standard deviation.

## 3. Results and Discussion

### 3.1. Performance of Fresh Concrete

#### 3.1.1. Setting Time

The setting time of fresh cement is given in Table 3. The addition of lignosulfonate, molasses raw materials, sugar solution, and filter mud from molasses postponed the setting of fresh cement, and the setting time gradually increased with the addition amount in the range of 0–0.8%; molasses raw materials, sugar solution and filter mud from molasses had a more significant influence on the retardation of fresh concrete than lignosulfonate; water soluble pigment had no significant influence on the setting time of fresh cement; molasses colloid had a significant influence on the retardation of fresh concrete.

Lignosulfonate is a commonly used cement retarder and water reducer, and its phenolic hydroxyl group and hydroxyl group participate in multiple chemical reactions and physical interactions in cement retardation. Hydrophobic frame C_6_-C_3_, sulfonic acid and other groups provide it with the structural characteristics of surfactant; thus, it shows certain surface physical and chemical properties, such as adsorption and dispersity, viscosity, rheological properties, and colloidal properties; however, it was a three-dimensional net structure of unsulfonated phenylpropane in the central site of the lignosulfonate molecule, and this kind of three-dimensional molecular structure hinders lignosulfonate from dispersing in cement; it was also detrimental to the adsorption of lignosulfonate on the surface of cement particles and failed to provide good adsorption retention and high surface activity, which caused poor compatibility between lignosulfonate and Portland cement [18]. Sugar solution, the source of molasses, mainly contains sucrose and a small amount of glucose and fructose [19]. These saccharides had good water solubility and good adsorption to cement particles [18]. When ordinary Portland cement hydrates in sugar solution, lime and sugar react to form chelates. These chelates could be adsorbed on the nucleation center of Ca(OH)_2_ and on C-S-H gels, thereby preventing cement from setting [20]; however, the addition amount of saccharides would affect the setting time of cement, and there was a critical dose of saccharides in retardation. On the one hand, when W/C was constant, the addition amount of saccharides was slightly lower than the critical dose, the formation of C-S-H was slow, and the setting time of cement was postponed.

When the addition amount exceeds the critical dose, the setting time could rapidly fall from the maximum until the coagulating effect occurs (Table 3) because excessive sugar accelerates the crystal formation of high-sulfur hydrated calcium sulfoaluminate (ettringite, AFt) with a coagulating effect [21]. On the other hand, the higher the *W/C* value was, the more pores in cement were to be filled; that is, more demand for solid hydrates results in a longer time for cement setting and hardening. When the retarder was added, the setting time was further increased, and the greater the addition amount was, the longer the setting time was [22]. When colloid was added alone, it shows an obvious coagulating effect (Table 3), but it could increase the dispersity of cement and achieve a water reducing effect [23]. The molasses raw materials contained 52.81% free sugar and 15.92% colloid. When molasses raw materials were used as exogenous admixtures, molasses showed both a retarding effect and a water reducing effect, and the colloid component with a good dispersity promoted saccharides and noncolloidal components to improve the retarding effect of cement [24]. Filter mud from molasses mainly contained free sugar, colloid and holocellulose, which were totally collected into the filter mud from molasses due to solid–liquid separation (Table 2). Celluloses are macromolecule polysaccharides composed of glucose, xylose and other monosaccharides that are nonionic compounds and have certain cement retarding performance (Table 2) and good water retaining performance. The retardation mechanism of celluloses could be explained according to Hansen adsorption theory: celluloses dispersed in water could be adsorbed on the surface of unhydrated cement particles, which hindered the initial invasion of water to cement particles, and thus, the hydration process was postponed, but it could be further explained according to Yong’s postponed nucleation theory: celluloses dispersed in water inhibit the formation and growth of crystal nuclei of Ca(OH)_2_; thus, the hydration process was postponed. In fact, the retarding effect of celluloses could be attributed to the combined effect of the above two theories [25]. In this study, filter mud from molasses effectively prolonged the initial setting time and final setting time of fresh cement concrete, which was attributed to the synergistic effect of holocellulose, sugar and colloid in filter mud. Under the condition that *W/C* and the added dose of admixtures were the same, the initial setting time and final setting time of cement with added filter mud from molasses advanced; however, increasing the amount of filter mud from molasses could reach the same effect as adding molasses raw materials, and in practical applications, the amount of filter mud from molasses could be selected as required; therefore, filter mud from molasses could still be considered an effective, promising cement retarder and a water reducer that could replace lignosulfonate, and it could achieve the purpose of making full use of molasses raw materials.

#### 3.1.2. Slump

The slump of the fresh concrete sample is given in Table 4.

As shown in Table 4, the greater the addition amount of retarder was, the larger the slump of fresh cement concrete was. When the addition amount of molasses, Filter mud and Ca-lignosulfonate increased from 0.2% to 0.4%, the air content in concrete increased from 0.5, 0.8 and 1.5 to 0.7, 1.0 and 1.8, respectively, and the slump increased from 14.8 cm, 15.7 cm and 17.0 cm to 18.7 cm, 19.9 cm and 21.8 cm, respectively. Meanwhile, under the conditions of the same *W/C* value and additional amount of retarder, the concrete with added lignosulfonate had the largest slump, that with added filter mud from molasses had the second largest slump, and that with added molasses raw materials had the smallest slump, which was probably attributed to the higher air content in fresh concrete with added lignosulfonate and that with added filter mud from molasses. Jumadurdiyev et al. [19] compared the influence of molasses and lignosulfonate on the slump of fresh concrete. When the addition amount of molasses and lignosulfonate increased from 0.25% to 0.50%, the air content in concrete increased from 0.3 and 1.6 to 0.6 and 2, respectively, and the slump increased from 14.5 cm and 17.5 cm to 18 cm and 20 cm, respectively. The air content could obviously change the workability of fresh concrete [20]. The slight difference in the slumps of the concretes in favour of the lignosulfonate-added one could be a result of the higher entrained air content of the latter concrete, because it is known that air entraining in concrete improves the workability, especially in lean mixes [21,22].

#### 3.1.3. Bleed Water

**Figure 2:**
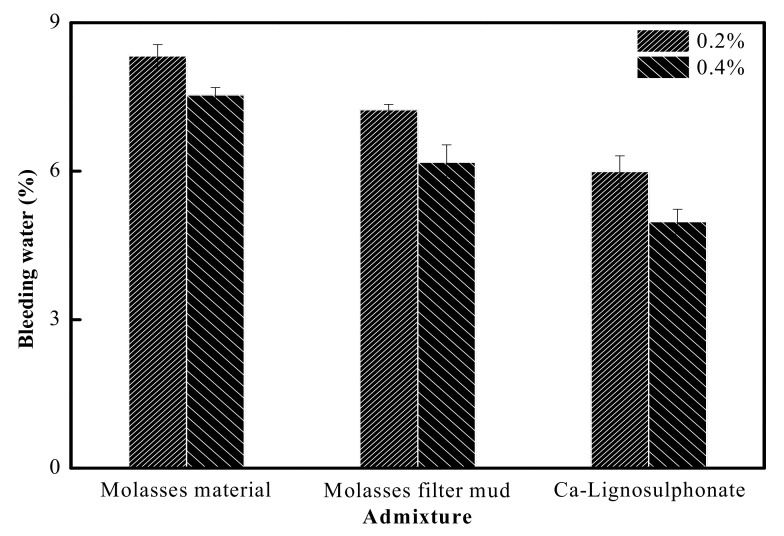
Effects of different additives on the total bleeding water of fresh cement.

The bleeding rates of fresh concrete with added molasses raw materials, filter mud from molasses, and lignosulfonate are given in Figure 2. The concrete with added molasses raw materials had the highest bleeding rate, that with added molasses had the second highest bleeding rate, and that with added lignosulfonate had the lowest bleeding rate; this was probably attributed to the higher air content in fresh concrete with added filter mud from molasses and that with added lignin sulfonate, and thus, the solid particles in the fresh cement remained floating, reduced the sedimentation of solid particles, disturbed the continuity of capillary pores in cement, and reduced the water seepage [15]. Meanwhile, the cellulose component had water retention performance, which could raise the viscosity and water retention of cement concrete and reduce the amount of bleed water [26].

### 3.2. Properties of Hardened Concrete

#### 3.2.1. Compressive Strength

**Figure 3:**
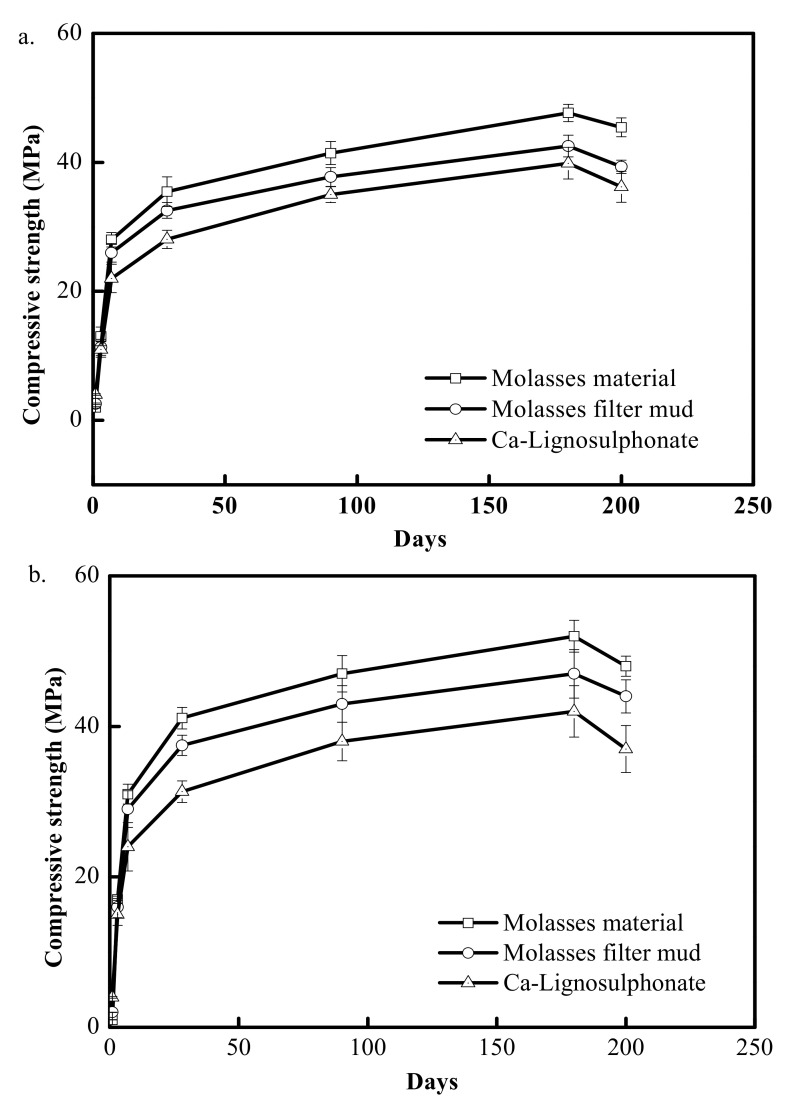
Effects of different additives on the cement compressive strength. (**a**) concrete with 0.2% retarder, (**b**) concrete with 0.4% retarder.

The influences of molasses raw materials, filter mud from molasses and lignin sulfonate on the compressive strength of hardened concrete are given in Figure 3. The compressive strength changed over time in additional amounts of 0.2% and 0.4%, as shown in Figure 3a,b, respectively. As shown in Figure 3, the compressive strength of concrete with retarder added was lower than that without retarder in the same time period because of the effect of retarder; however, over time, the compressive strength of concrete with retarder could reach and even exceed that without retarder, and the compressive strengths of concrete with molasses and filter mud from molasses were greater than the compressive strength of concrete with lignin sulfonate. For instance, after hardening for 28 days, the concrete with 0.2% and 0.4% molasses raw materials had the highest compressive strength of 35.46 MPa and 41.11 MPa accordingly. The concrete with 0.2% and 0.4% lignosulfonate had 28.07 MPa and 31.33 MPa, respectively, but those with 0.2% and 0.4% filter mud from molasses had a compressive strength of 32.55 MPa and 37.48 MPa of compressive strength accordingly. The compressive strength of concrete with 0.2% and 0.4% filter mud from molasses was 15.96% and 19.62% higher, respectively, than that with lignosulfonate. The concrete with added molasses and that with added filter mud from molasses had higher compressive strength, which was probably attributed to a homogeneous and compact structure formed in the hardening process [27]; however, the compressive strength of concrete with retarder added declined on the 200th day. For the concrete with added sugar at the age between 180 d and 365 d, a similar trend was observed [19], which was probably attributed to the shrinkage crack incurred after concrete hardening [22]; nevertheless, the concrete with added filter mud from molasses also had a higher compressive strength than that with added lignosulfonate.

#### 3.2.2. Water Absorption

Water absorption of concrete with 0.4% molasses, 0.4% molasses and lignosulfonate was observed (as shown in Table 5), and the amount of water absorbed per unit area of capillary in 60 min from the concrete was measured at the age of 180 days. When cumulative water absorption was expressed by the square root of time, Formula (2) was obtained [11]:

(2) $$Q=a+K\sqrt{t}$$

where *Q* is the cumulative water absorption per unit area, mm; a is a constant, min; and *K* is the absorbance.

As shown in Table 5, the cement concrete sample with lignosulfonate added had the poorest water absorption capacity, only 0.164 mm/min, but the cement concrete with molasses and filter mud from molasses added had better water absorption capacities, 0.238 mm/min and 0.221, 45.12% and 34.76% higher than that with lignosulfonate added, respectively. Jumadurdiyev et al. [19] compared the water absorption capacities of concrete with added molasses and that with added lignosulfonate and found that the water absorption amount of concrete with added molasses was 0.20–0.25 mm/min and that of the concrete with added lignosulfonate was 0.186 mm/min. The results of this study indicated that when the *W/C* value was the same, the concrete with added lignosulfonate and that with added filter mud from molasses had poorer water absorption capacity than that with added molasses, which was attributed to the higher air content in the concrete with added lignosulfonate and that with added filter mud from molasses. In most cases, bubbles in the concrete reduced the number of pores and disturbed the continuity of capillary pores in the cement. In addition, the cement concrete supplemented with lignosulfonate and that supplemented with molasses showed a lower bleeding rate than that supplemented with molasses; this was because the concrete with added lignosulfonate and that with added molasses had poorer water absorption capacity than that with added molasses.

#### 3.2.3. Drying Shrinkage

**Figure 4:**
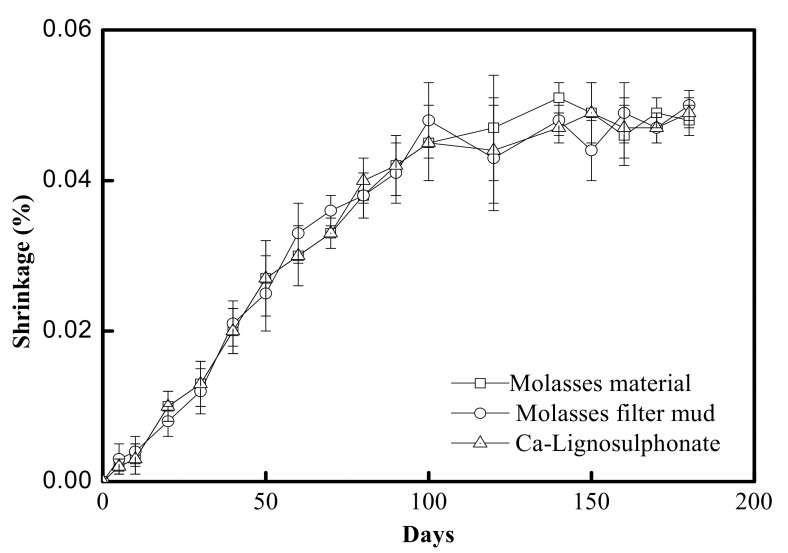
Monitoring drying shrinkage of concretes for admixture content of 0.4%.

The results of the drying shrinkage test of concrete at the age of 0 to 180 days are given in Figure 4. A similar process of drying shrinkage was shown in the concrete with added molasses raw materials, that with added filter mud from molasses and that with added lignosulfonate. Sugar, molasses raw materials and filter mud from molasses could be used as a coating retarder. Although the flame retardant mixture ionised in the stirred water of concrete increases the drying shrinkage rate, the coating type had no effect on the drying shrinkage [23,26].

#### 3.2.4. Freezing and Thawing Properties

**Figure 5:**
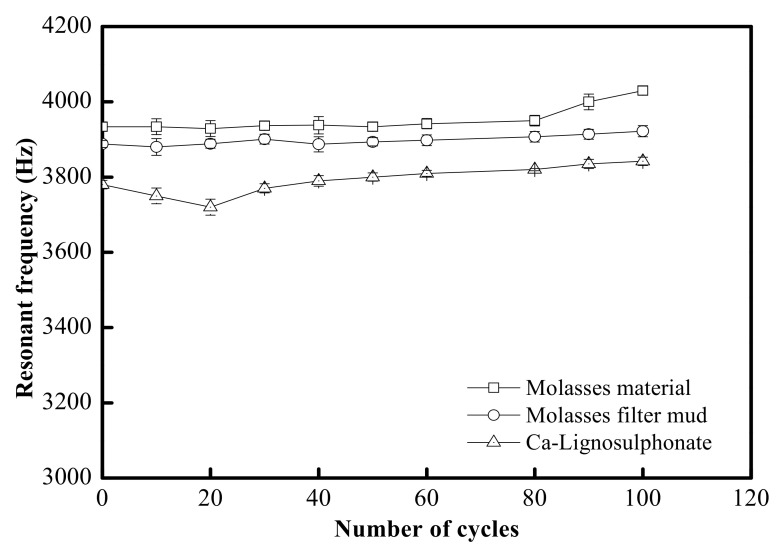
Variation of resonant frequency with freezing–thawing cycles.

The variation in resonant frequency in the freezing–thawing test was 100 cycles. As shown in Figure 5, in terms of the resonant frequency value, 100 cycles of freezing–thawing did not show an adverse effect on concrete. Ashworth [8] compared the freezing–thawing resistance of concrete with different *W/C* values and found that the concrete with *W/C* equal to or higher than 0.60 had good freezing–thawing resistance, and the concrete with added sugar had better freezing–thawing resistance than that without sugar; however, after 100 cycles, the measured longitudinal ultrasonic velocity showed that the cement concrete sample had suffered slight deterioration (Table 6). The velocity decreased by 6.33% in the concrete with added molasses, by 7.10% in the concrete with added filter mud from molasses, and by 12.54% in the concrete with added lignosulfonate. Jumadurdiyev et al. [19] composed the freezing–thawing resistance after 140 cycles of freezing–thawing tests and found that the longitudinal ultrasonic velocity in the concrete with added molasses decreased by 7% to 9%, and the mass decreased by 0.5% to 0.6%, whereas for the concrete with added lignosulfonate, a 0.6% reduction occurred in longitudinal ultrasonic velocity and some reduction in mass; therefore, it could be concluded that after 100 cycles of the freezing–thawing test, the concrete with added molasses had the best freezing–thawing resistance; compared to the concrete with added lignosulfonate, the concrete with added filter mud from molasses had better freezing–thawing resistance.

#### 3.2.5. Wetting and Drying Properties

**Figure 6:**
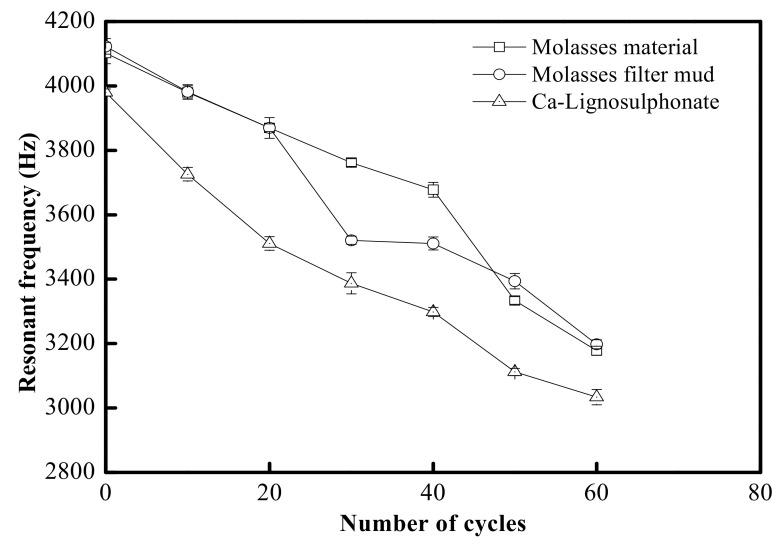
Variation of resonant frequency with wetting–drying cycles.

After 60 cycles of the wetting and drying test, the concrete sample obtained resonant frequency and ultrasonic velocity, as shown in Figure 6 and Table 7. After the initial hardening of 28 days, the variation trends in the resonant frequency and ultrasonic velocity of the concrete with added molasses raw materials, that with added filter mud from molasses and that with added lignosulfonate were consistent, which indicated that all these kinds of concrete were adversely affected by the wetting-drying cycle. Jumadurdiyev et al. [19] compared the influence of molasses and lignosulfonate on the wetting and drying properties of concrete after 60 cycles of freezing–thawing and found that the longitudinal ultrasonic velocity in the concrete with added molasses decreased by 13% to 16%, and the mass decreased by 1.7% to 1.9%, whereas for the concrete with added lignosulfonate, a 17% reduction occurred in longitudinal ultrasonic velocity and a 2.4% reduction in mass; therefore, compared to the results of the freezing–thawing test, wetting and drying were more likely to damage the quality of the concrete with added filter mud from molasses and other retarders.

#### 3.2.6. Carbonisation

**Figure 7:**
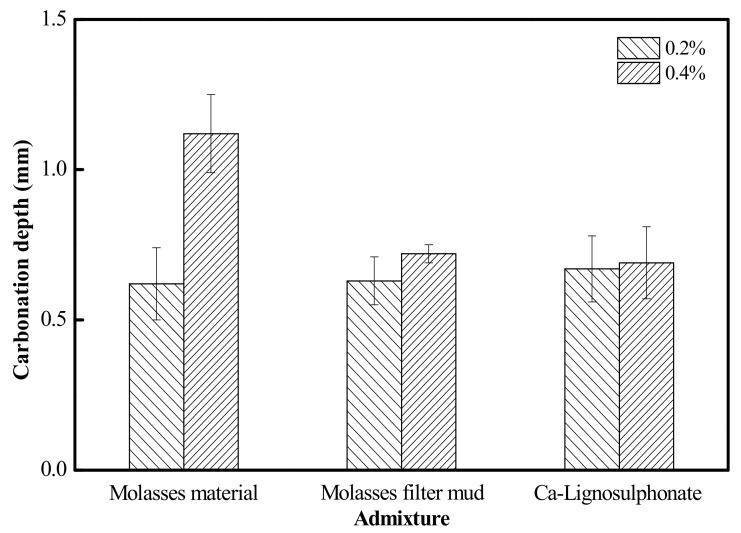
Effects of admixture on carbonation depth.

The carbonation depth of the concrete with added molasses raw materials, that with added filter mud from molasses and that with added lignin sulfonate is shown in Figure 7. Molasses raw materials, filter mud from molasses and lignin sulfonate were added to cement with addition amounts of 0.2% and 0.4%, respectively. The results of hardened cement concrete at the age of 180 days indicated that the larger the addition amount of molasses raw materials was, the deeper the carbonation depth of concrete was; however, for the concrete with added filter mud from molasses and that with added lignin sulfonate, the addition amount had no significant influence on the carbonation depth. Jumadurdiyev et al. [19] composed the carbonation depth of cement concrete with added molasses and that with added lignin sulfonate from different sources and found that when the addition amount of lignosulfonate was between 0.25% and 0.5%, the carbonation depth remained the same; therefore, it could be concluded that the concrete with added filter mud from molasses and that with added lignosulfonate show similar properties in carbonation depth, and filter mud from molasses could be used as an alternative admixture for lignosulfonate.

## 4. Conclusions

The main conclusions can be summarised from this work as follows. Molasses filter mud could be used as a good substitute for lignosulfonate, and could also partially replace molasses as retarder and water reducer of OPC. The molasses filter mud-added cement pastes showed expanded setting times even in 0.2% dosage; however, the water-soluble pigment in molasses had no significant effect on the setting time of cement paste, and colloids significantly promoted the setting of cement paste. In addition, the filter mud significantly reduced the slump of cement concrete and increased the compressive strength and water absorption capacity of OPC. After 100 freeze–thaw cycles, the concrete with molasses filter mud had better freeze-thaw resistance than the concrete with lignosulfonate; therefore, the performance of filter mud in concrete workability was similar to that of lignosulfonate-based admixture, which provided a potential way for the comprehensive utilisation of molasses.

## Figures and Tables

**Table 1:** Properties and composition of cement.

Physical Properties	Mechanical Properties
Density (g/m^3^) 3.15	Compressive strength/MPa 3 d 25.4 7 d 35.8 28 d 46.3
Blaine sp. surface (cm^2^/g) 3900	Chemical components/% C_3_S (Tricalcium silicate) 52.74 C_2_S (Dicalcium silicate) 15.63 C_3_A (Tricalcium aluminate) 4.01 C_4_AF (Fe-Aluminate) 13.22
Grade Degree of fineness (80 μm) 3.8% Degree of fineness (30 μm) 10.9%	
Setting time (min) Beginning time 150 ± 10 Final setting time 265 ± 10	

**Table 2:** The distribution of molasses components.

Composition	Molasses Material/%	Filter Mud/%	Molasses Colloids/%	Sugar Liquid/%
Free sugar	52.81 ± 0.02	3.98 ± 0.18	-- ^a^	40.60 ± 2.45
Colloids	15.92 ± 1.25	2.25 ± 0.08	12.85 ± 1.14	--
Holocellulose	3.22 ± 0.14	3.22 ± 0.58	--	--
Ash	5.05 ± 0.79	1.75 ± 0.67	1.36 ± 0.02	1.23 ± 0.11

^a^—indicated “no detection”.

**Table 3:** Effects of different additives on cement condensation time.

Admixture Ratio	0%		0.2%		0.4%		0.8%	
	Start/min	End/min	Start/min	End/min	Start/min	End/min	Start/min	End/min
Ca-lignosulfonate	150 ± 10	265 ± 10	210 ± 8	311 ± 10	282 ± 5	363 ± 13	413 ± 12	538 ± 3
Molasses material	150 ± 10	265 ± 10	454 ± 11	558 ± 6	732 ± 13	985 ± 17	920 ± 11	1258 ± 12
Water-soluble pigment	150 ± 10	265 ± 10	150 ± 2	265 ± 12	153 ± 8	267 ± 9	155 ± 11	272 ± 9
Sugars	150 ± 10	265 ± 10	421 ± 12	500 ± 10	673 ± 2	863 ± 22	888 ± 21	1098 ± 28
Sucrose	150 ± 10	265 ± 10	423 ± 23	503 ± 2	688 ± 12	877 ± 12	900 ± 12	1197 ± 20
Molasses colloids	150 ± 10	265 ± 10	113 ± 17	228 ± 19	98 ± 10	189 ± 18	65 ± 11	132 ± 10
Filter mud in molasses	150 ± 10	265 ± 10	298 ± 11	416 ± 7	485 ± 12	738 ± 21	752 ± 14	1072 ± 16

**Table 4:** Workability properties and air contents of fresh concretes.

Admixture Content/%	Molasses Material	Filter Mud	Ca-Lignosulfonate
Fresh concrete/cm			
0.2	14.8 ± 0.2	15.7 ± 0.3	17.0 ± 0.3
0.4	18.7 ± 0.2	19.9 ± 0.4	21.8 ± 0.5
Air contents/%			
0.2	0.5 ± 0.05	0.8 ± 0.03	1.4 ± 0.05
0.4	0.7 ± 0.02	1.0 ± 0.05	1.8 ± 0.03

**Table 5:** Sorptivities of concretes.

Condition	Sorptivity (mm/min)		
	Molasses Material	Filter Mud	Ca-Lignosulfonate
28 d in water	0.238 ± 0.15	0.221 ± 0.12	0.164 ± 0.08

**Table 6:** The changes of ultrasound velocity and weight of concretes under freezing–thawing cycles.

		Admixture		
		Molasses Material	Filter Mud	Ca-Lignosulfonate
Ultrasound velocity(km/s)	Initial	4216 ± 20	4223 ± 12	4219 ± 15
	After 100 cycles	3949 ± 15	3923 ± 21	3690 ± 7
	Change/%	6.33 ± 0.25	7.10 ± 1.33	12.54 ± 0.98
Weight of specimen/g	Initial	12,230 ± 25	12,246 ± 23	12,126 ± 12
	After 100 cycles	12,143 ± 11	12,146 ± 17	12,035 ± 24
	Change/%	0.71 ± 0.11	0.82 ± 0.13	0.75 ± 0.22

**Table 7:** The changes of ultrasound velocity and weight of concretes under wetting–drying cycles.

		Admixture		
		Molasses Material	Filter Mud	Ca-Lignosulfonate
Ultrasound velocity (km/s)	Initial	4423 ± 14	4435 ± 21	4322 ± 32
	After 60 cycles	3776 ± 12	3679 ± 35	3580 ± 23
	Change/%	14.63 ± 1.35	17.05 ± 2.11	17.17 ± 2.41
Weight of specimen/g	Initial	12,318 ± 25	12,352 ± 23	12,245 ± 12
	After 60 cycles	12,103 ± 25	12,188 ± 22	11,947 ± 14
	Change/%	1.75 ± 0.14	1.32 ± 0.24	2.43 ± 0.15

## Data Availability

Not applicable.
